# Supplementary material for: Regulation Effectiveness and Mechanism of Biotransformation Pathway on the Toxicity of Microcystin-LR Target to Protein Phosphatase 2A
Source: Int J Environ Res Public Health. 2023 Jan 5;20(2):964. doi: 10.3390/ijerph20020964 (PMC9859369; doi:10.3390/ijerph20020964)
Supplement: Supplementary file 1 [file ijerph-20-00964-s001.zip › ijerph-1961854-supplementary.pdf]

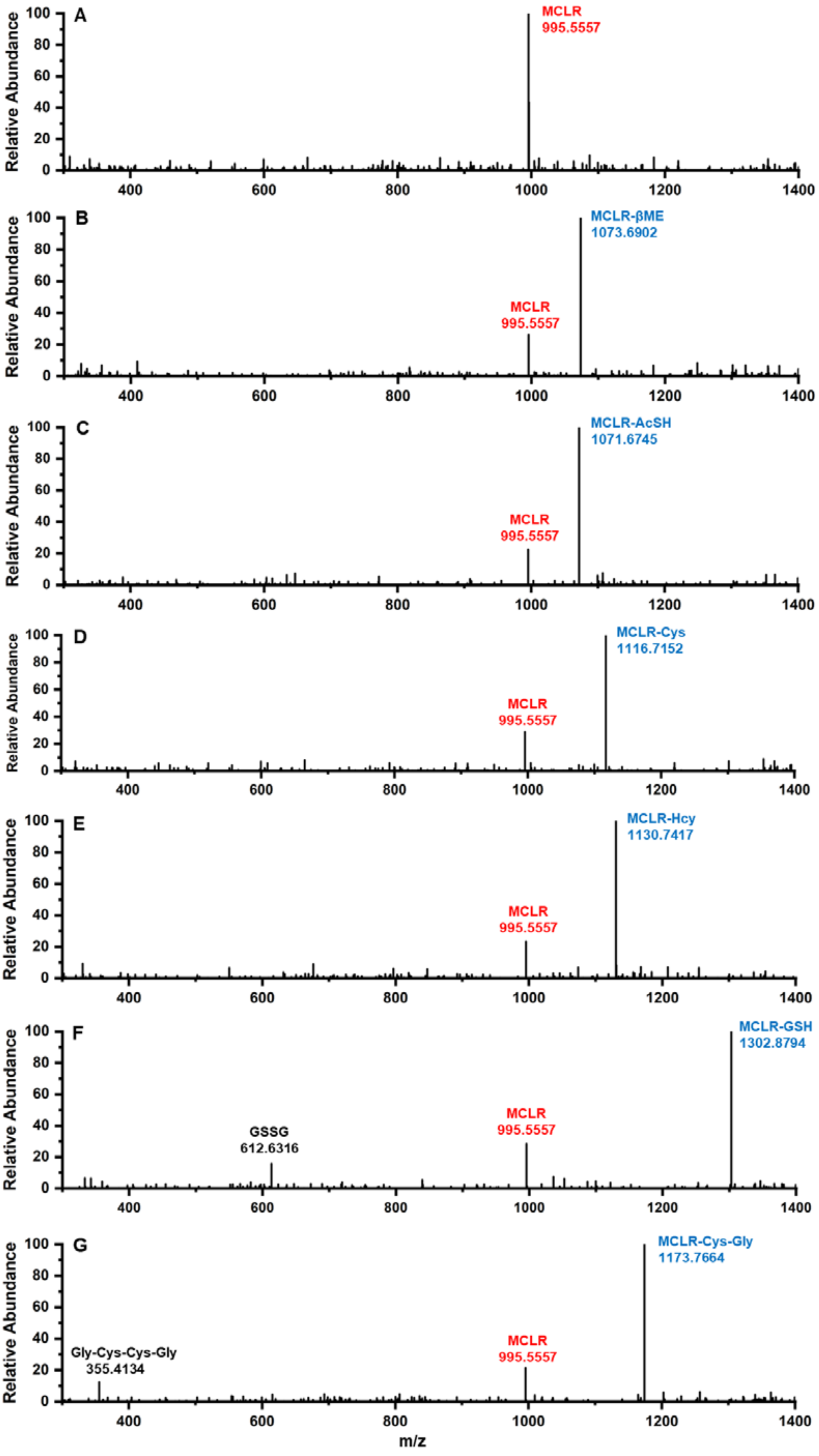
A
MCLR
995.5557
B
MCLR-βME
1073.6902
MCLR
995.5557
C
MCLR-AcSH
1071.6745
MCLR
995.5557
D
MCLR-Cys
1116.7152
MCLR
995.5557
E
MCLR-Hcy
1130.7417
MCLR
995.5557
F
MCLR-GSH
1302.8794
GSSG
612.6316
MCLR
995.5557
G
MCLR-Cys-Gly
1173.7664
Gly-Cys-Cys-Gly
355.4134
MCLR
995.5557
Relative Abundance
m/z

**Figure S1.** The MS analysis for MCLR (A), and its electrophilic addition samples with the βME (B), AcSH (C), Cys (D), Hcy (E), GSH (F), and Cys-Gly (G).

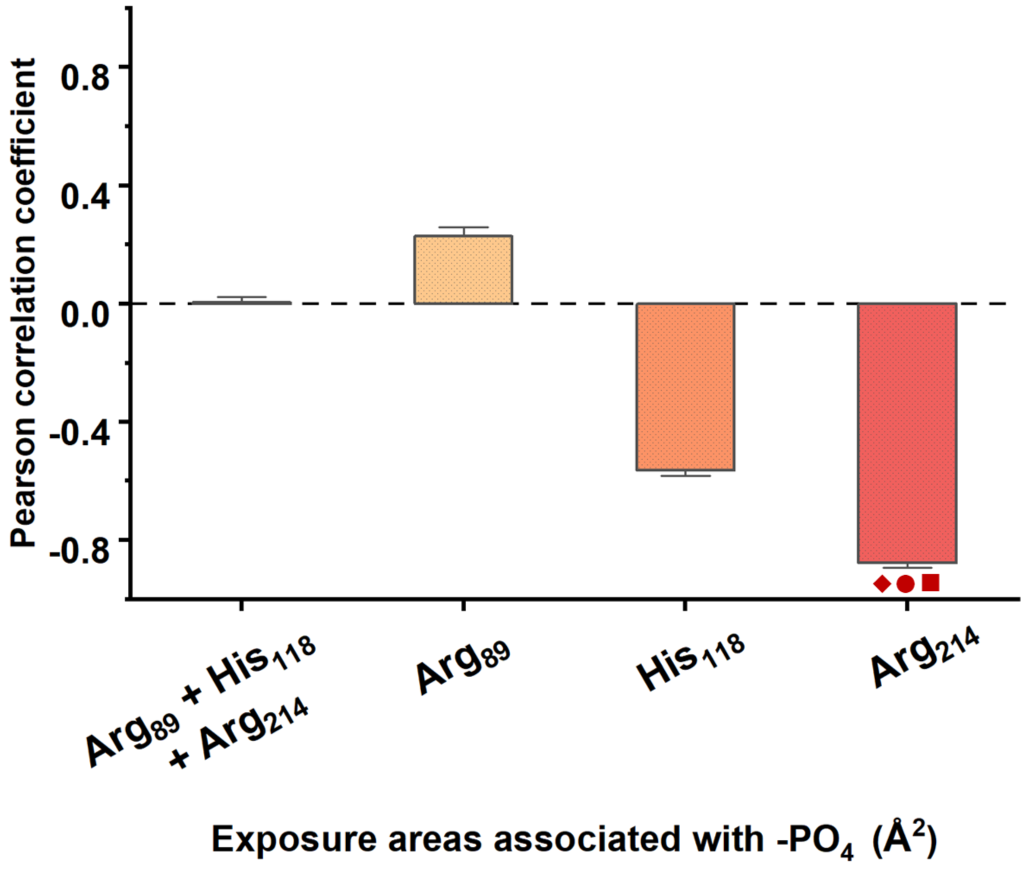


**Figure S2.** Pearson correlation coefficients between inhibition data and the exposure areas associated with phosphate group.

**Table S1.** MS/MS identification of MCLR and MCLR-BTPs.

| Basic fragment ions | MCLR | MCLR-βME | MCLR-AcSH | MCLR-Cys | MCLR-Hcy | MCLR-GSH | MCLR-Cys-Gly |
|---|---|---|---|---|---|---|---|
| $[M + H]^+$ | 995.5557 | 1073.6902 | 1071.6745 | 1116.7152 | 1130.7417 | 1302.8794 | 1173.7664 |
| | | (+ 78.1345)↑[a] | (+ 76.1188)↑ | (+ 121.1595)↑ | (+ 135.1860)↑ | (+ 307.3237)↑ | (+ 178.2107)↑ |
| $[PhCH_2CH(OCH_3)]^+$ | 135.0803 | 135.0803√[b] | 135.0802√ | 135.0804√ | 135.0803√ | 135.0801√ | 135.0804√ |
| $[Glu\text{-}Mdha+H]^+$ | 213.0832 | 291.2175↑ | 289.2017↑ | 334.2424↑ | 348.2693↑ | 520.4070↑ | 391.2939↑ |
| $[MeAsp\text{-}Arg+H]^+$ | 286.1477 | 286.1476√ | 286.1477√ | 286.1478√ | 286.1476√ | 286.1479√ | 286.1479√ |
| $[Mdha\text{-}Ala\text{-}Leu\text{-}MeAsp+H]^+$ | 397.2079 | 475.3422↑ | 473.3265↑ | 518.3674↑ | 532.3937↑ | 704.5315↑ | 575.4186↑ |
| or $[Glu\text{-}Mdha\text{-}Ala+Leu+H]^+$ | | | | | | | |
| $[Arg\text{-}Adda+H]^+$ | 470.3124 | 470.3123√ | 470.3122√ | 470.3124√ | 470.3125√ | 470.3123√ | 470.3122√ |
| $[Mdha\text{-}Ala\text{-}Leu\text{-}MeAsp\text{-}Arg+H]^+$ | 553.3072 | 631.4416↑ | 629.4258↑ | 674.4664↑ | 688.4931↑ | 860.6307↑ | 731.5179↑ |
| $[MeAsp\text{-}Arg\text{-}Adda+H]^+$ | 599.3542 | 599.3541√ | 599.3542√ | 599.3545√ | 599.3541√ | 599.3542√ | 599.3543√ |
| or $[Arg\text{-}Adda\text{-}Glu+H]^+$ | | | | | | | |
| $[Arg\text{-}Adda\text{-}Glu\text{-}Mdha+H]^+$ | 682.3957 | 760.5304↑ | 758.5143↑ | 803.5554↑ | 817.5818↑ | 989.7195↑ | 860.6065↑ |
| $[Mdha\text{-}Ala\text{-}Leu\text{-}MeAsp\text{-}Arg\text{-}Adda+H]^+$ | 866.5149 | 944.6493↑ | 942.6333↑ | 987.6744↑ | 1001.7010↑ | 1173.8387↑ | 1044.7254↑ |
| or $[Arg\text{-}Adda\text{-}Glu\text{-}Mdha\text{-}Ala\text{-}Leu+H]^+$ | | | | | | | |
| Target residues | --- | Mdha | | | | | |

[a:] ↑ and ↓ mean mass changes were related to these fragment ions; [b:] √ means ions with the stable m/z were detected by mass spectrograph.

**Table S2.** Preparation and purification information for the electrophilic addition samples of MCLR.

| **Conjugation products** | **Eluted time**[a] | **Concentration**[b] | **Total volume** | **Purity**[c] |
|---|---|---|---|---|
| MCLR-βME | 19.7 min | ≈1200 μmol/L | 5 × 100 μL | 98.3% |
| MCLR-AcSH | 20.1 min | ≈1000 μmol/L | 5 × 100 μL | 97.2% |
| MCLR-Cys | 17.4 min | ≈1100 μmol/L | 5 × 100 μL | 98.9% |
| MCLR-Hcy | 17.3 min | ≈1100 μmol/L | 5 × 100 μL | 98.0% |
| MCLR-GSH | 12.5 min | ≈1200 μmol/L | 5 × 100 μL | 98.5% |
| MCLR-Cys-Gly | 15.8 min | ≈1200 μmol/L | 5 × 100 μL | 96.7% |

[a] Collection time was set for 0.5 min (± 0.25 min around the eluted time).

[b] With 200 μmol/L MCLR served as the inner standard for quantification and assumed MCLR and MCLR-BTPs had approximate protonated efficiencies.

[c] Purity was directed calculated by the MS signals of MCLR and MCLR-BTPs.

**Table S3.** The candidate interaction parameters between MCLR/MCLR-BTPs and PP2A.

| Molecular simulation parameters | | MCLR | MCLR-βME | MCLR-Acsh | MCLR-Cys | MCLR-Hcy | MCLR-GSH | MCLR-Cys-Gly |
|---|---|---|---|---|---|---|---|---|
| Combination area (Å$^2$) | **Total** | 683.0694 | 716.4927 | 728.2102 | 716.6841 | 703.6324 | 788.2092 | 763.7774 |
| | Ala$^1$→PP2A | 50.2139 | 18.2943 | 11.9711 | 14.6462 | 13.6280 | 12.0700 | 11.3981 |
| | Leu$^2$→PP2A | 135.0409 | 125.4982 | 140.9633 | 123.6076 | 119.4272 | 134.4739 | 134.4101 |
| | MeAsp$^3$→PP2A | 59.4977 | 58.7240 | 69.3921 | 67.4127 | 66.9446 | 69.8673 | 68.8829 |
| | Arg$^4$→PP2A | 115.7080 | 112.9425 | 110.6440 | 113.6880 | 111.2893 | 108.8118 | 108.4528 |
| | Adda$^5$→PP2A | 360.9873 | 359.5962 | 359.7151 | 358.5792 | 358.0810 | 355.9954 | 356.1675 |
| | Glu$^6$→PP2A | 171.7180 | 160.6913 | 168.2875 | 160.7650 | 159.8249 | 161.6061 | 165.1835 |
| | Mdha$^7$→PP2A | 120.6914 | 171.9261 | 156.0190 | 183.8890 | 175.3485 | 228.8137 | 208.1651 |
| Positive accessible surface area (Å$^2$) | **Total** | 438.9368 | 463.9523 | 456.8462 | 456.5521 | 456.1923 | 495.0612 | 496.7734 |
| | Ala$^1$→PP2A | 25.0459 | 17.2165 | 24.2321 | 4.6460 | 3.6280 | 27.6002 | 26.9446 |
| | Leu$^2$→PP2A | 90.8749 | 82.9635 | 91.6720 | 79.6961 | 79.5110 | 87.1274 | 87.1095 |
| | MeAsp$^3$→PP2A | 32.0448 | 31.4304 | 38.9354 | 38.3280 | 31.1080 | 38.5582 | 30.9814 |
| | Arg$^4$→PP2A | 64.9071 | 64.5195 | 74.2696 | 77.0562 | 78.1798 | 72.6489 | 74.0464 |
| | Adda$^5$→PP2A | 232.0883 | 230.8065 | 236.1999 | 235.9119 | 238.0377 | 236.7063 | 236.5531 |
| | Glu$^6$→PP2A | 94.3617 | 92.7170 | 92.8100 | 92.5818 | 91.9039 | 90.0932 | 90.3951 |
| | Mdha$^7$→PP2A | 84.1601 | 111.4355 | 117.9418 | 141.2325 | 131.8605 | 195.7521 | 141.3499 |
| Negative accessible surface area (Å$^2$) | **Total** | 244.1328 | 252.5401 | 271.3640 | 263.7243 | 258.1291 | 293.1485 | 267.0042 |
| | Ala$^1$→PP2A | 15.1677 | 1.0778 | 7.7387 | 3.8035 | 13.4443 | 3.4700 | 4.4534 |
| | Leu$^2$→PP2A | 44.1661 | 42.5345 | 49.2910 | 47.3340 | 50.6234 | 47.3470 | 47.3030 |
| | MeAsp$^3$→PP2A | 27.4573 | 27.2935 | 30.4565 | 32.6770 | 39.5256 | 30.9170 | 29.9012 |
| | Arg$^4$→PP2A | 37.8009 | 38.4228 | 37.3740 | 41.2243 | 48.7987 | 36.4634 | 36.4063 |
| | Adda$^5$→PP2A | 124.0795 | 125.1887 | 121.8808 | 126.2599 | 132.3664 | 122.8900 | 122.4342 |
| | Glu$^6$→PP2A | 77.3566 | 67.9743 | 71.4769 | 71.7758 | 78.6100 | 71.5129 | 71.7884 |

| | | | | | | | | |
|---|---|---|---|---|---|---|---|---|
| | Mdha$^{7}$→PP2A | 36.5312 | 42.2267 | 38.8030 | 39.9037 | 41.1499 | 41.3724 | 41.4820 |
| Hydrophobic surface area (Å$^{2}$) | **Total** | 404.7893 | 408.4791 | 408.6372 | 401.2150 | 408.7348 | 405.9594 | 405.5256 |
| | Ala$^{1}$→PP2A | 8.6175 | 2.2335 | 3.6345 | 1.2400 | 0.3619 | 4.8209 | 4.6412 |
| | Leu$^{2}$→PP2A | 59.2876 | 52.8599 | 62.9961 | 49.6412 | 46.6786 | 56.7017 | 56.2078 |
| | MeAsp$^{3}$→PP2A | 6.6383 | 4.8506 | 8.0488 | 8.3986 | 8.2832 | 8.0563 | 8.9237 |
| | Arg$^{4}$→PP2A | 39.8851 | 41.3816 | 38.9211 | 41.0544 | 41.8457 | 36.9658 | 37.8200 |
| | Adda$^{5}$→PP2A | 245.7937 | 242.2990 | 242.9248 | 244.8030 | 240.0145 | 238.1998 | 238.0276 |
| | Glu$^{6}$→PP2A | 49.8923 | 45.4832 | 48.0880 | 46.1070 | 42.9830 | 40.8250 | 43.5837 |
| | Mdha$^{7}$→PP2A | 87.8027 | 101.4919 | 93.2629 | 95.9085 | 98.9036 | 91.4386 | 90.7020 |
| Polar surface area (Å$^{2}$) | **Total** | 278.2801 | 308.0134 | 319.5731 | 315.4689 | 294.8981 | 377.2498 | 351.2517 |
| | Ala$^{1}$→PP2A | 41.5964 | 16.0608 | 28.3363 | 3.4061 | 3.2663 | 26.2488 | 26.7570 |
| | Leu$^{2}$→PP2A | 75.7535 | 72.6383 | 77.9671 | 73.9661 | 72.7487 | 77.7724 | 78.2022 |
| | MeAsp$^{3}$→PP2A | 52.8644 | 53.8734 | 61.3445 | 59.0138 | 58.6617 | 61.4187 | 59.9589 |
| | Arg$^{4}$→PP2A | 62.8229 | 61.5608 | 72.7224 | 73.6337 | 74.4438 | 72.1461 | 72.6327 |
| | Adda$^{5}$→PP2A | 118.1400 | 117.7955 | 115.0663 | 138.7763 | 113.7906 | 64.2971 | 113.1936 |
| | Glu$^{6}$→PP2A | 121.8257 | 115.2084 | 120.1989 | 114.6581 | 116.8472 | 120.7811 | 121.5998 |
| | Mdha$^{7}$→PP2A | 32.8887 | 70.4345 | 73.7561 | 87.9808 | 67.4454 | 136.3751 | 115.4629 |
| Active center exposure (Å$^{2}$) | $Mn_1^{2}$+$Asp_{57}$+$Asp_{85}$+$Asn_{117}$+$His_{167}$+$His_{241}$ | 819.0067 | 819.8222 | 821.4860 | 823.0130 | 822.2631 | 824.9606 | 822.1924 |
| | $Mn_1^{2+}$+$Asp_{57}$ | 314.8653 | 317.4326 | 317.4961 | 317.9027 | 318.2437 | 317.3058 | 317.6216 |
| | $Mn_1^{2+}$+$Asp_{85}$ | 310.2860 | 306.5590 | 307.7299 | 307.0732 | 307.6104 | 309.5630 | 307.9066 |
| | $Mn_1^{2+}$+$Asn_{117}$ | 320.5904 | 321.9788 | 321.9153 | 321.3859 | 321.5473 | 321.5898 | 321.8677 |
| | $Mn_1^{2+}$+$His_{241}$ | 356.1933 | 354.9973 | 355.6072 | 354.4759 | 356.6136 | 354.5811 | 356.0070 |
| | $Mn_2^{2+}$+$Asp_{57}$+ $His_{59}$+$Asp_{85}$ | 562.7151 | 558.7158 | 558.6845 | 562.9078 | 570.1757 | 568.3002 | 562.5989 |
| | $Mn_2^{2+}$+$His_{59}$ | 347.0402 | 342.9848 | 338.8241 | 346.8755 | 347.2521 | 348.9503 | 347.8009 |

| | | | | | | | | |
|---|---|---|---|---|---|---|---|---|
| | $Mn_2^{2+}+Asp_{85}$ | 316.8060 | 316.4693 | 315.4002 | 316.2285 | 316.6199 | 316.9627 | 315.1873 |
| Exposure areas associated with $-PO_4$ (Å$^2$) | $Arg_{89}+His_{118}+Arg_{214}$ | 1001.8087 | 1005.6117 | 927.2913 | 924.2706 | 922.9355 | 1000.8712 | 1000.4306 |
| | $Arg_{89}$ | 374.1155 | 375.9034 | 375.4064 | 375.9305 | 376.6839 | 372.9275 | 375.3104 |
| | $His_{118}$ | 319.8264 | 318.1479 | 320.9894 | 319.5455 | 319.8376 | 319.6681 | 321.7038 |
| | $Arg_{214}$ | 322.0467 | 375.2818 | 374.9326 | 373.0033 | 371.4669 | 372.916 | 373.6877 |
| Hydrogen bond (KJ/Mol) | **Total** | -44.3 | -37 | -42 | -42.2 | -35.4 | -50.9 | -49.9 |
| | $Pro_{213} \leftarrow Arg^{4}$ | -7 | -6.7 | -6.5 | -6.5 | -6.5 | -6.5 | -6.7 |
| | $Arg_{268} \rightarrow Ala^{1}$ | -2.6 | ---[a] | --- | --- | --- | --- | --- |
| | $Arg_{89} \rightarrow Leu^{2}$ | -2.2 | -2.6 | -2.3 | -4.3 | -4.1 | -1.9 | -2.6 |
| | $Arg_{89} \rightarrow MeAsp^{3}$ | -6.8 | -5.8 | -6.8 | -4.1 | -3.9 | -7.2 | -6.3 |
| | $Arg_{214} \rightarrow Arg^{4}$ | -6.6 | -5.2 | -5.1 | -4.9 | -4.9 | -5.3 | -5.3 |
| | $Asn_{117} \rightarrow Adda^{5}$ | -0.9 | -2.6 | -1 | -2.3 | -2.5 | --- | --- |
| | $His_{118} \rightarrow Adda^{5}$ | -1.7 | -1.5 | -1.5 | -1.5 | -1.5 | -1.5 | -1.4 |
| | $Arg_{89} \rightarrow Glu^{6}$ | -8.2 | -1.6 | -8.1 | -0.5 | --- | -7.5 | -7.4 |
| | $Arg_{268} \leftarrow Mdha^{7}$ | -6.7 | -6.8 | -9.2 | -8.8 | -8.9 | -13.4 | -13.7 |
| | $Arg_{268} \rightarrow Mdha^{7}$ | --- | -1.9 | --- | -6.6 | -1.5 | -2.2 | -4.1 |
| | $Tyr_{127} \rightarrow MeAsp^{3}$ | -1.6 | --- | -1.5 | -1.5 | -1.6 | -1.5 | -1.6 |
| | $Cys_{269} \rightarrow Mdha^{7}$ | --- | --- | --- | -1.2 | --- | -1.1 | -0.8 |
| | $Gly_{270} \rightarrow Mdha^{7}$ | --- | --- | --- | --- | --- | -2.8 | --- |
| | $Cys_{266} \rightarrow Mdha^{7}$ | --- | -2.3 | --- | --- | --- | --- | --- |
| Ionic bond (KJ/Mol) | **Total** | -110.4 | -106.6 | -109.5 | -104.7 | -104 | -109.7 | -109 |
| | $Arg_{89}-MeAsp^{3}$ | -7.5 | -7.7 | -7.7 | -6.8 | -6.9 | -7.9 | -7.6 |
| | $Arg_{89}-Glu^{6}$ | -6.9 | -3.2 | -5.9 | -2.3 | -1.7 | -6.5 | -6.3 |
| | $Mn_1^{2+}-Glu^{6}$ | -12.2 | -12.2 | -12.1 | -12.1 | -12.1 | -11.9 | -11.8 |
| | $Mn_2^{2+}-Glu^{6}$ | -22.5 | -22.4 | -22.8 | -22.4 | -22.3 | -22.6 | -22.8 |

| | | | | | | | | |
|---|---|---|---|---|---|---|---|---|
| | $Asp_{57}$-$Mn_1^{2+}$ | -12.2 | -12.1 | -11.9 | -12.1 | -11.9 | -11.9 | -11.9 |
| | $Asp_{57}$-$Mn_2^{2+}$ | -15.3 | -15.5 | -15.4 | -15.5 | -15.6 | -15.4 | -15.2 |
| | $Asp_{85}$-$Mn_1^{2+}$ | -21.6 | -21.5 | -21.5 | -21.5 | -21.5 | -21.3 | -21.2 |
| | $Asp_{85}$-$Mn_2^{2+}$ | -12.2 | -12 | -12.2 | -12 | -12 | -12.2 | -12.2 |
| Metal bond (KJ/Mol) | **Total** | -42.8 | -42.6 | -42.1 | -42.5 | -42.1 | -42.5 | -42.6 |
| | $Mn_1^{2+}$-$Glu^6$ | -4.6 | -4.7 | -4.5 | -4.7 | -4.5 | -4.5 | -4.3 |
| | $Mn_2^{2+}$-$Glu^6$ | -9.2 | -9 | -9 | -8.9 | -8.7 | -9.1 | -9.1 |
| | $Asp_{57}$-$Mn_1^{2+}$ | -5.3 | -5.2 | -5.1 | -5.2 | -5.1 | -5 | -5.1 |
| | $Asp_{57}$-$Mn_2^{2+}$ | -5.7 | -5.8 | -5.8 | -5.8 | -5.8 | -5.7 | -5.7 |
| | $Asp_{85}$-$Mn_1^{2+}$ | -6.8 | -6.5 | -6.3 | -6.6 | -6.8 | -6.7 | -6.8 |
| | $Asp_{85}$-$Mn_2^{2+}$ | -4.8 | -4.8 | -4.9 | -4.9 | -4.8 | -4.8 | -4.8 |
| | $His_{241}$-$Mn_1^{2+}$ | -2 | -2.2 | -2.2 | -2.1 | -2.1 | -2.4 | -2.6 |
| | $Asn_{117}$-$Mn_1^{2+}$ | -4.4 | -4.4 | -4.3 | -4.3 | -4.3 | -4.3 | -4.2 |

[a]: **---** no related parameter was detected
